# Accuracy of abbreviated protocols for unattended automated office blood pressure measurements, a retrospective study

**DOI:** 10.1371/journal.pone.0248586

**Published:** 2021-03-15

**Authors:** Annina S. Vischer, Rebecca Hug, Thenral Socrates, Andrea Meienberg, Michael Mayr, Thilo Burkard

**Affiliations:** 1 Medical Outpatient Department and Hypertension Clinic, ESH Hypertension Centre of Excellence, University Hospital Basel, Basel, Switzerland; 2 Department of Cardiology, University Hospital Basel, Basel, Switzerland; Toyama Daigaku, JAPAN

## Abstract

**Background:**

Blood pressure measurement (BPM) is one of the most often performed procedures in clinical practice, but especially office BPM is prone to errors. Unattended automated office BPM (AOBPM) is somewhat standardised and observer-independent, but time and space consuming. We aimed to assess whether an AOBPM protocol can be abbreviated without losing accuracy.

**Design:**

In our retrospective single centre study, we used all AOBPM (AOBPM protocol of the SPRINT study), collected over 14 months. Three sequential BPM (after 5 minutes of rest, spaced 2 minutes) were automatically recorded with the patient alone in a quiet room resulting in three systolic and diastolic values. We compared the mean of all three (RefProt) with the mean of the first two (ShortProtA) and the single first BPM (ShortProtB).

**Results:**

We analysed 413 AOBPM sets from 210 patients. Mean age was 52±16 years. Mean values for RefProt were 128.3/81.3 mmHg, for ShortProtA 128.4/81.4 mmHg, for ShortProtB 128.8/81.4 mmHg. Mean difference and limits of agreement for RefProt vs. ShortProtA and ShortProtB were -0.1±4.2/-0.1±2.8 mmHg and -0.5±8.1/-0.1±5.3 mmHg, respectively. With ShortProtA, 83% of systolic and 92% of diastolic measurements were within 2 mmHg from RefProt (67/82% for ShortProtB). ShortProtA or ShortProtB led to no significant hypertensive reclassifications in comparison to RefProt (p-values 0.774/1.000/1.000/0.556).

**Conclusion:**

Based on our results differences between the RefProt and ShortProtA are minimal and within acceptable limits of agreement. Therefore, the automated procedure may be shorted from 3 to 2 measurements, but a single measurement is insufficient.

## 1 Introduction

Arterial hypertension (AHT) has a very high and at least in low- and middle-income countries still increasing prevalence with 31% of the world adult population affected in the year 2010 [1]. AHT is one of the leading causes for premature death by causing both haemorrhagic and ischaemic stroke, myocardial infarction, heart failure, peripheral artery disease and end-stage renal disease [2–5]. For proper AHT detection and treatment it is essential that blood pressure (BP) is measured correctly [6]. Despite the importance of correct BP assessment, office blood pressure measurement (OBPM) is often performed incorrectly in daily practice [7,8].

Automated office blood pressure measurement (AOBPM) uses fully automated oscillometric programmed devices on a patient sitting alone in a quiet room to measure BP according to a given protocol, which is, however, not fully standardized. Protocols are usually device-related and differ in the pretest rest phase, the test rest phase, the numbers of measurements taken and the time between measurements [9]. A commonly applied protocol uses the mean of three repeated measurements after 5 minutes of rest. AOBPM gained special attention after its use in the SPRINT (Systolic Blood Pressure Intervention Trial) and is the preferred method for OBPM according to the Hypertension Canada Guidelines [10,11]. This method is able to overcome multiple aspects of human error occurring during OBPM such as single readings, conversation with patients (if the patient is not in the same room with the health professional), and digit preference, and may reduce a “white coat effect” resulting in lower values than OBPM [9,12,13]. AOBPM correlates better with day-time ambulatory blood pressure monitoring (ABPM) and home blood pressure measurements (HBPM) than OBPM [9,14]. AOBPM can improve the diagnosis of AHT in office, and therefore reduce overtreatment and overcome other limitations of OBPM [14,15], though the method is discussed controversially because of an apparent higher variability in comparison to ABPM [16,17]. It has been criticized, that AOBPM may increase costs in comparison to OBPM due to the required space and staff needed [18].

The aim of this retrospective study was to assess whether an AOBPM protocol can be abbreviated without losing accuracy.

## 2 Methods

### 2.1 Patient population and recruitment

We conducted a retrospective analysis by chart review between 1 January 2017 and 28 February 2018, as our clinic started examining patients with AOBPM on single days at that time. For this purpose, we collected the AOBPM results from all patients measured by AOBPM at the Hypertension Clinic at the University Hospital Basel during this time period. For patients with multiple appointments during this period, we included the measurements from all appointments. Only complete measurements following a standardized procedure were included in the study.

### 2.2 Automated blood pressure measurement procedure

The patients were sitting upright with the back supported and uncrossed legs in a quiet room by themselves. The arm rested stretched and supported on an arm rest. The correct cuff was selected to match the arm circumference and was positioned at the level of the heart [5,19]. The nurse performed a test measurement to check if the device was working properly, then started the test sequence and left the room. During the recorded measurement itself, the device automatically took three measurements after 5, 7 and 9 minutes. We used a Welch Allyn Connex® Spot Monitor which applies the SureBP® measurement technique by Welch Allyn, which has been validated according to the American National Standards Institute/Association for the Advancement of Medical Instrumentation SP10:2006 (AAMI) and the British Hypertension Society (BHS) 1993 protocols [20]. The monitor was programmed for the reference unattended automated blood pressure measurement algorithm, i.e. measurements after 5, 7 and 9 minutes.

### 2.3 Documented parameters

We collected all three BP readings and date of the measurement, as well as age, sex, height and weight of the patient and the number of consultations. Additional data, such as cardiovascular risk factors and pre-existing conditions like pregnancy, coronary heart disease, heart failure, arterial hypertension, diabetes mellitus, peripheral artery disease, renal failure, transient ischemic attack /stroke and smoking status as well as antihypertensive medication, were also collected and transferred anonymously into predefined Case Report Files (CRF).

### 2.4 Calculation of blood pressure values

Based on the three single BP values (labelled S1, S2, S3 and D1, D2, D3, respectively), we calculated the mean of all three measurements (RefProt), which we used as the reference BP for each visit. As the test BP, we used the mean of the first and the second measurement (ShortProtA) and the single first measurement (ShortProtB) for each patient and visit.

### 2.5 Classification of arterial hypertension and blood pressure ranges

The systolic and diastolic values from RefProt, ShortProtA and ShortProtB were classified as normotensive or hypertensive. Patients with a systolic BP ≥ 135 mmHg or diastolic BP ≥ 85 mmHg were regarded as hypertensive, all other values as normotensive. This cutoff is recommended in the Hypertension Canada guidelines [11] to correlate with the traditional values of ≥ 140/90 mmHg, as recommended for OBPM by the European Society of Hypertension and Cardiology (ESC/ESH) guidelines [5]. This value is derived from the cutoff value for daytime ABPM, which is considered the best correlation for all AOBPM measurement protocols [9,11,14]. Systolic and diastolic values were classified separately. In addition, all BP values were classified in ranges, with a low range defined as < 120 / <70 mmHg, intermediate range as 120–160 / 70–100 mmHg, and high range >160 / >100 mmHg, adapted according to ESC/ESH guidelines. We handled systolic and diastolic values separately, also for these ranges.

### 2.6 Statistical analyses

Continuous data were reported as mean ± standard deviation (SD) if normally distributed or otherwise as median (inter-quartile range (IQR)). The Shapiro-Wilk-Test was used to test for normality. We tested for association using the related-samples Wilcoxon signed rank test, for which we calculated the effect size using Rosenthal’s formula [21], and Bland-Altman plots and for linearity by making scatter plots. We ran an exact McNemar’s test in case of ≤ 25 discordant pairs and a McNemar’s test with continuity correction in case of > 25 discordant pairs to determine if there were differences in BP classifications with either procedure.

All statistical analyses were performed using SPSS version 22 and R version 3.6.0.

### 2.7 Ethical approval and trial registration

This trial was approved by the local ethics committee (EKNZ2018-00295; Ethikkommission Nordwest- und Zentralschweiz/Ethics Committee Northwestern and Central Switzerland, Basel, Switzerland, eknz@bs.ch), was compliant with the Declaration of Helsinki. Written informed consent was waived by the local ethics committee for this retrospective patient data analysis.

## 3 Results

### 3.1 Baseline characteristics

We included measurements from 210 patients resulting in 413 complete AOBPM sets. 96 patients had one, 58 two, 33 three, 15 four, 6 five, and 2 patients had six AOBPM sets. Baseline characteristics are shown in S1 Table. 55 measurements (13.3%) were taken without antihypertensive medication.

### 3.2 Blood pressure values

Median and mean values for the RefProt, ShortProtA and ShortProtB and the number of AOBPM sets in each range are shown in Table 1. Smooth density plots depicting a range of 84–208 mmHg for the systolic RefProt values and 55–113 mmHg for the diastolic RefProt values are shown in S1 Fig.

**Table 1:** Median (inter-quartile range (IQR)) and mean (± standard deviation (SD)) blood pressure value in mmHg and blood pressure range according to each protocol.

Protocol	Median (IQR)	Mean (± SD)	Low range, n (%)	Intermediate range, n (%)	High range, n (%)
**syst. RefProt**	127.7 (116.8–139.2)	128.3 (± 17.5)	128 (31.0)	269 (65.1)	16 (3.9)
**syst. ShortProtA**	127.5 (117.0–140.0)	128.4 (± 17.7)	129 (31.2)	269 (65.1)	15 (3.6)
**syst. ShortProtB**	128.0 (117.0–142.0)	128.8 (± 18.4)	133 (32.2)	262 (63.4)	18 (4.4)
**diast. RefProt**	80.3 (75.7–86.5)	81.3 (± 10.0)	42 (10.2)	353 (85.5)	18 (4.4)
**diast. ShortProtA**	80.0 (75.5–86.5)	81.4 (± 10.2)	41 (9.9)	353 (85.5)	19 (4.6)
**diast. ShortProtB**	80.0 (75.0–86.5)	81.4 (± 10.7)	42 (10.2)	349 (84.5)	22 (5.3)

Low range: < 120 / <70 mmHg, intermediate range: 120–160 / 70–100 mmHg, high range: >160 / >100 mmHg. RefProt: Mean of all three measurements. ShortProt: ShortProtA: Mean of the first two measurements, ShortProtB: The single first measurement.

### 3.3 Correlation of blood pressure values

#### 3.3.1 Systolic values

There was no statistically significant median difference between the RefProt and the ShortProtA (p-value 0.203), but between the RefProt and the ShortProtB (p-value 0.021). The median differences and ranges are shown in Table 2. A Bland-Altman plot showed no systematic error in S2 Fig, panel A (ShortProtA), and no systematic error, but wider limits of agreement, in panel B (ShortProtB). The number of measurements with an absolute difference between RefProt and ShortProtA and ShortProtB, respectively <2, <5 and <10 mmHg can be found in S2 Table.

**Table 2:** Median difference between RefProt, ShortProt A and ShortProtB displayed as median and range.

Test BP	Median difference RefProt–ShortProt (mmHg)	Range difference RefProt–ShortProt (mmHg)	z-value	p-value	Test effect
**syst. ShortProtA**	- 0.2	-7.7–7.8	-1.27	0.203	0.063
**syst. ShortProtB**	- 0.3	-12.7–13.3	-2.32	0.021	0.114
**diast. ShortProtA**	- 0.2	-7.7–5.8	-2.05	0.040	0.101
**diast. ShortProtB**	- 0.3	-16.3–16.3	-0.96	0.335	0.047

RefProt: Mean of all three measurements. ShortProt: ShortProtA: Mean of the first two measurements, ShortProtB: The single first measurement.

#### 3.3.2 Diastolic values

There was a statistically significant median difference between the RefProt and the ShortProtA (p-value 0.040), but not between the RefProt and the ShortProtB (p-value 0.335). The median differences and ranges are shown in Table 2. A Bland-Altman plot is showing no systematic error in S2 Fig, panel C (ShortProtA), and no systematic error, but wider limits of agreement, in panel D (ShortProtB). The number of measurements with an absolute difference between RefProt and ShortProtA and ShortProtB, respectively <2, <5 and <10 mmHg can be found in S2 Table.

### 3.4 Linear relationship between blood pressure values

**Fig 1:**
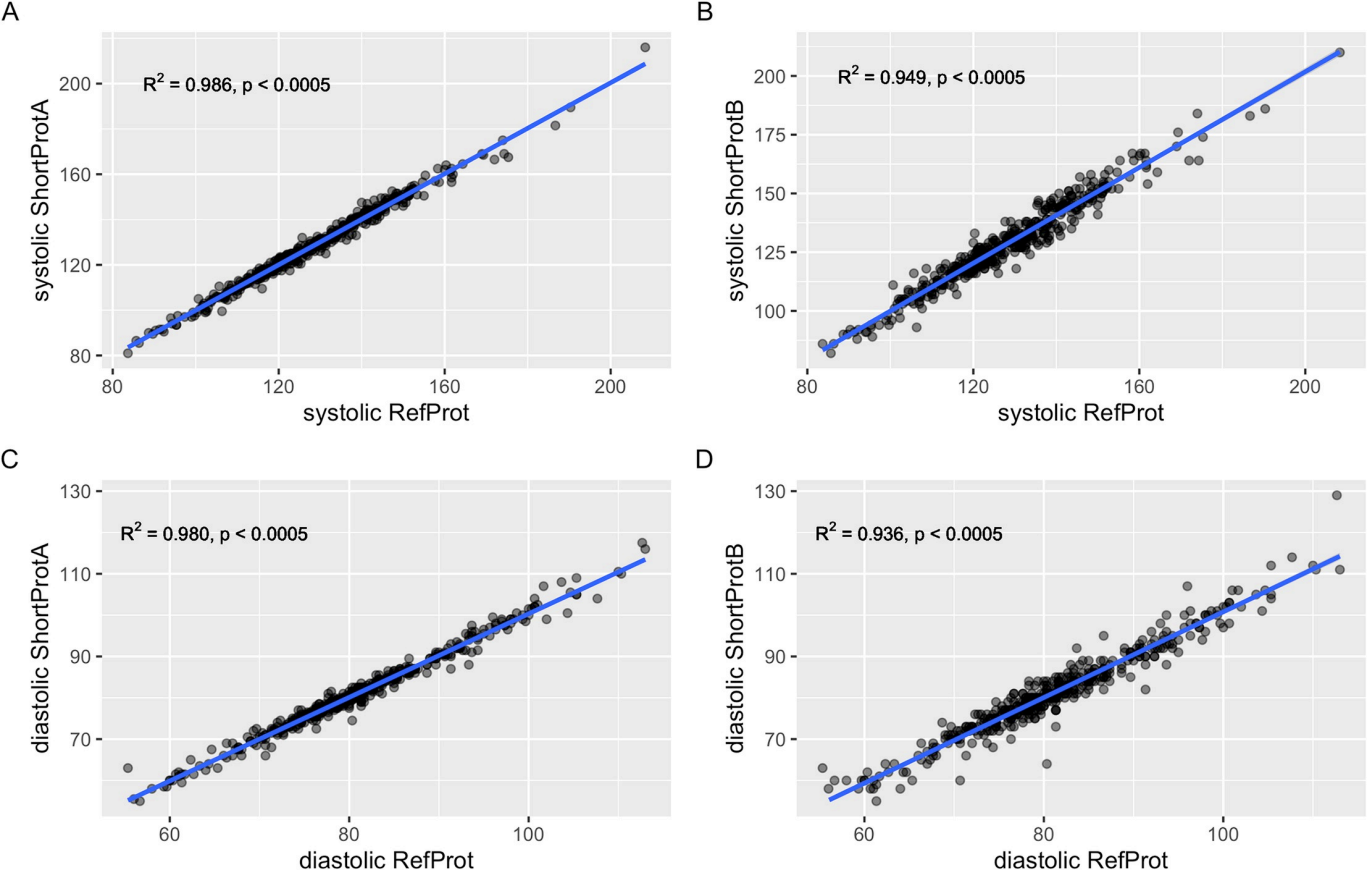
Scatter plots with regression lines comparing systolic RefProt to ShortProtA (panel A), and with ShortProtB (panel B); and diastolic RefProt to ShortProtA (panel C), and ShortProtB (panel D).

Scatterplots show a linear relationship between the systolic values from RefProt and ShortProtA (Fig 1, panel A) and ShortProtB (Fig 1, panel B). Similar results are seen for the diastolic values from RefProt and ShortProtA (Fig 1, panel C) and ShortProtB (Fig 1, panel D).

### 3.5 Blood pressure classification

#### 3.5.1 Systolic values

Of all 413 AOBPM sets 141 (34.1%) were classified as hypertensive by applying the RefProt procedure. Applying the ShortProtA resulted in no statistically significant difference in hypertensive classifications (139 AOBPM sets, 33.7%, p = 0.774). Applying the ShortProtB resulted in no statistically significant difference in hypertensive classifications (140 AOBPM sets, 33.9%, p = 1.000). Reclassifications for each of the 413 comparisons of RefProt with ShortProtA and ShortProtB are shown in S3A and S3B Fig, demonstrating more patients reclassified from hypertensive to normotensive and vice versa by ShortProtB than by ShortProtA. See also S3 Table for exact numbers.

#### 3.5.2 Diastolic values

Regarding diastolic values, of all 413 AOBPM sets 126 (30.5%) were classified as hypertensive by applying the RefProt procedure. Applying the ShortProtA resulted in no statistically significant difference in hypertensive classifications (126 AOBPM sets, 30.5%, p = 1.000). Applying the ShortProtB resulted in no statistically significant difference in hypertensive classification (130 AOBPM sets, 31.4%, p = 0.556). Reclassifications for each of the 413 comparisons of RefProt with ShortProtA and ShortProtB are shown in S3C and S3D Fig, demonstrating more patients reclassified from hypertensive to normotensive and vice versa by ShortProtB than by ShortProtA. See also S3 Table for exact numbers.

## 4 Discussion

The present analysis shows that a standard AOBPM protocol using the mean of three consecutive measurements, hence similar to the one used in the SPRINT trial and to the Omron HEM 907 AOBPM protocol, can be abbreviated to the use of two measurements without losing accuracy. We observed no clinically relevant BP differences on a global level and no relevant number of re-classifications on an individual measurement level between the AOBPM protocols studied. This result is delectable considering that the mean RefProt BP was 128/81 mmHg and therefore relatively close to the hypertensive cutoff. Bland Altman-plots show excellent limits of agreement for ShortProtA, but not for ShortProtB. Additionally, there was a very high level of agreement with 83% of systolic and 92% of diastolic measurements within two and 99% and 100% of systolic and diastolic measurements, respectively, within five mmHg [22]. A single unattended measurement (ShortProtB), in contrast, is not enough, as we found only 67/85% of the systolic measurements and 82/97% of the diastolic measurements within two and five mmHg respectively.

On the first thought, the simplest way to assess BP seems to be OBPM. However, as our group has shown previously, there are variations between the recommendations for the procedure between guidelines, which can affect BP values as well as classification [19,23,24]. OBPM are prone to mistakes in the procedure such as not waiting for 5 minutes, talking, or taking only one measurement [7,25]. Furthermore, if BP is measured correctly by a physician or nurse, this consumes at least nine minutes of time of the doctor or nurse and, when applying ESH guidelines, even 11 min in approximately 30% of patients [5]. During these 9 to 11 minutes patient and observer should be calm without interaction, who hence is bound and cannot complete any other tasks [26]. By using AOBPM a single nurse can measure more than one patient in parallel, in our clinic up to four patients at a time.

Both ABPM and HBPM outperform OBPM in prediction of cardiovascular death [27,28]. However, ABPM is uncomfortable and therefore usually not preferred by the patients, especially due to fears of interrupted sleep [29]. Concerns about HBPM include a lack of specific training for patients and thus inadequate measurements, and a reporting bias [30]. An alternative is to take unattended, i.e. AOBPM. Compared to other office BPM methods, AOBPM results in lower values than OBPM and correlates best with daytime ABPM [14,15]. AOBPM is most prominently recommended by the Hypertension Canada Guidelines [11]. Though previous versions recommended using a specific device, which takes six readings at a time, the most recent Hypertension Canada guidelines have deleted this recommendation [11,31,32]. The first measurement should be taken by a health professional to verify cuff position and validity of the measurement [11,31]. The 2016 Hypertension Canada guidelines recommended to use the next five measurements to calculate the average, which represents the AOBPM result [31]. The latest guidelines, however, no longer recommend a specific number of measurements or a specified rest period before the first measurement [11]. The impact of the AOBPM protocol on the comparability with ABPM is unclear, as the meta-analyses comparing these two modalities included studies with a variety of protocols [9,14]. The largest study to date that applied AOBPM was the SPRINT study [10]. In this study, they used three readings at 2-minute intervals after 5 min of rest with a specifically programmed Omron device (HEM-907) [33], as we do in our hypertension clinic but using an alternative device. With the availability of programmable monitoring devices nowadays, it is getting easier to implement AOBPM in clinical practice, as the BpTRU™ device is no longer available.

Concerns have been raised, that the introduction of AOBPM into daily clinical practice would require more time for clinic visits and room in order to keep patients undisturbed [18]. Whereas it has been shown that medical staff can undertake other clinical task while patients are undergoing AOBPM, the requirements in at least some space remain undisputable [26]. Though AOBPM can also be taken in a quiet waiting room, there is still a requirement in space and tranquillity but it gives the possibility to define a quiet blood pressure measurement room [34]. An abbreviated two-measurement-protocol might therefore help to implement AOBPM, by mitigating two of the usual reservations against AOBPM, which are the need of time and space.

The abbreviated AOBPM protocol with two measurements after a waiting time of 5 minutes, results, as shown in our data, in no significant differences in comparison to a standard AOBPM using the mean of 3 measurements.

### 4.1 Limitations

This is a retrospective study. As we have not assessed the outcome of the included patients, we are unable to the validity for outcome prediction with either protocol. The lack of data regarding correlation of AOBPM with outcome is generally critizised [18,35], though SPRINT and ACCORD are large and well known outcome studies applying AOBPM [10,36] and in addition, some smaller studies have been published [37].

## 5 Conclusion

Based on our results differences between the reference procedure using the mean of three unattended BPM and an abbreviated protocol using the mean of only two unattended BPM are minimal and within acceptable limits of agreement. Therefore, the AOBPM procotol could possibly be shortened to two measurements. A single unattended measurement, however, is insufficient.

## Supporting information

S1 Fig Smooth density plots for systolic (panel A) and diastolic (panel B) blood pressure values. (DOCX)

S2 Fig Bland-Altman plots comparing systolic RefProt to ShortProtA (panel A), and ShortProtB (panel B); and diastolic RefProt to ShortProtA (panel C), and ShortProtB (panel D). (DOCX)

S3 Fig Comparison of systolic BP classification of RefProt and ShortProtA (panel A), ShortProtB (panel B), and diastolic BP classification of RefProt and ShortProtA (panel C), and ShortProtB (panel D). One stethoscope equals to one AOBPM set. Pink: RefProt and ShortProt normotensive, Blue: RefProt normotensive, ShortProt hypertensive, Orange: RefProt hypertensive, ShortProt normotensive, Green: RefProt and ShortProt hypertensive.
(DOCX)

S1 Table Baseline characteristics. (DOCX)

S2 Table Number of measurements with an absolute difference <2, <5, <10 and <15 mmHg between the RefProt and ShortProtA and ShortProtB, respectively). (DOCX)

S3 Table BP classification of ShortProt in comparison to RefProt. (DOCX)
