# Supplementary material for: Accuracy of abbreviated protocols for unattended automated office blood pressure measurements, a retrospective study
Source: PLoS One. 2021 Mar 15;16(3):e0248586. doi: 10.1371/journal.pone.0248586 (PMC7959338; doi:10.1371/journal.pone.0248586)
Supplement: S1 Fig — (DOCX) [file pone.0248586.s001.docx]

**Supporting Figure S1:** **Smooth density plots for systolic (panel A) and diastolic (panel B) blood pressure values.**


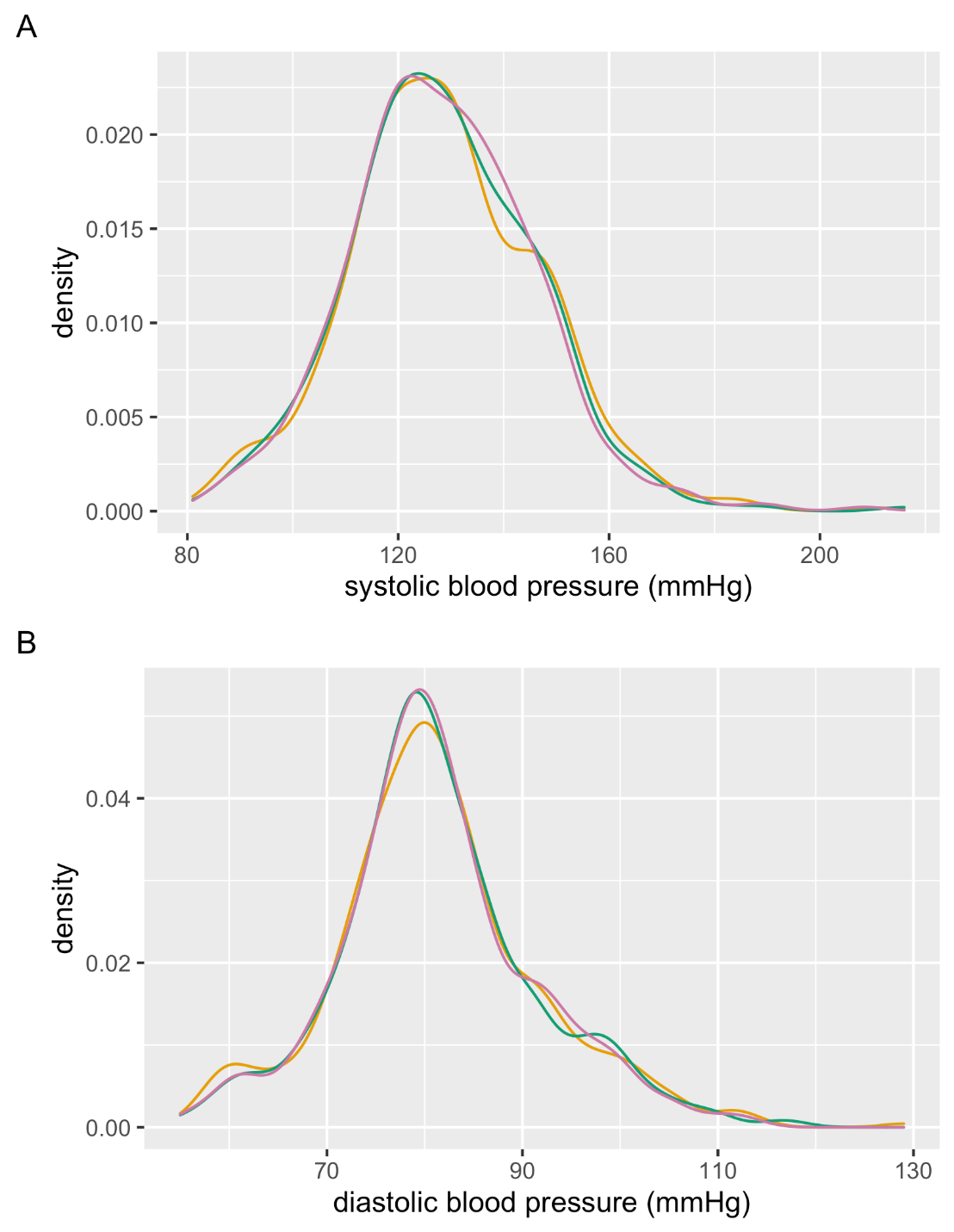


Reddish purple line: RefProt, green line: ShortProtA, orange line: ShortProtB.
