# Supplementary material for: Accuracy of abbreviated protocols for unattended automated office blood pressure measurements, a retrospective study
Source: PLoS One. 2021 Mar 15;16(3):e0248586. doi: 10.1371/journal.pone.0248586 (PMC7959338; doi:10.1371/journal.pone.0248586)
Supplement: S2 Fig — (DOCX) [file pone.0248586.s002.docx]

**Supporting Figure S2: Bland-Altman plots comparing systolic RefProt to ShortProtA (panel A), and ShortProtB (panel B); and diastolic RefProt to ShortProtA (panel C), and ShortProtB (panel D).**


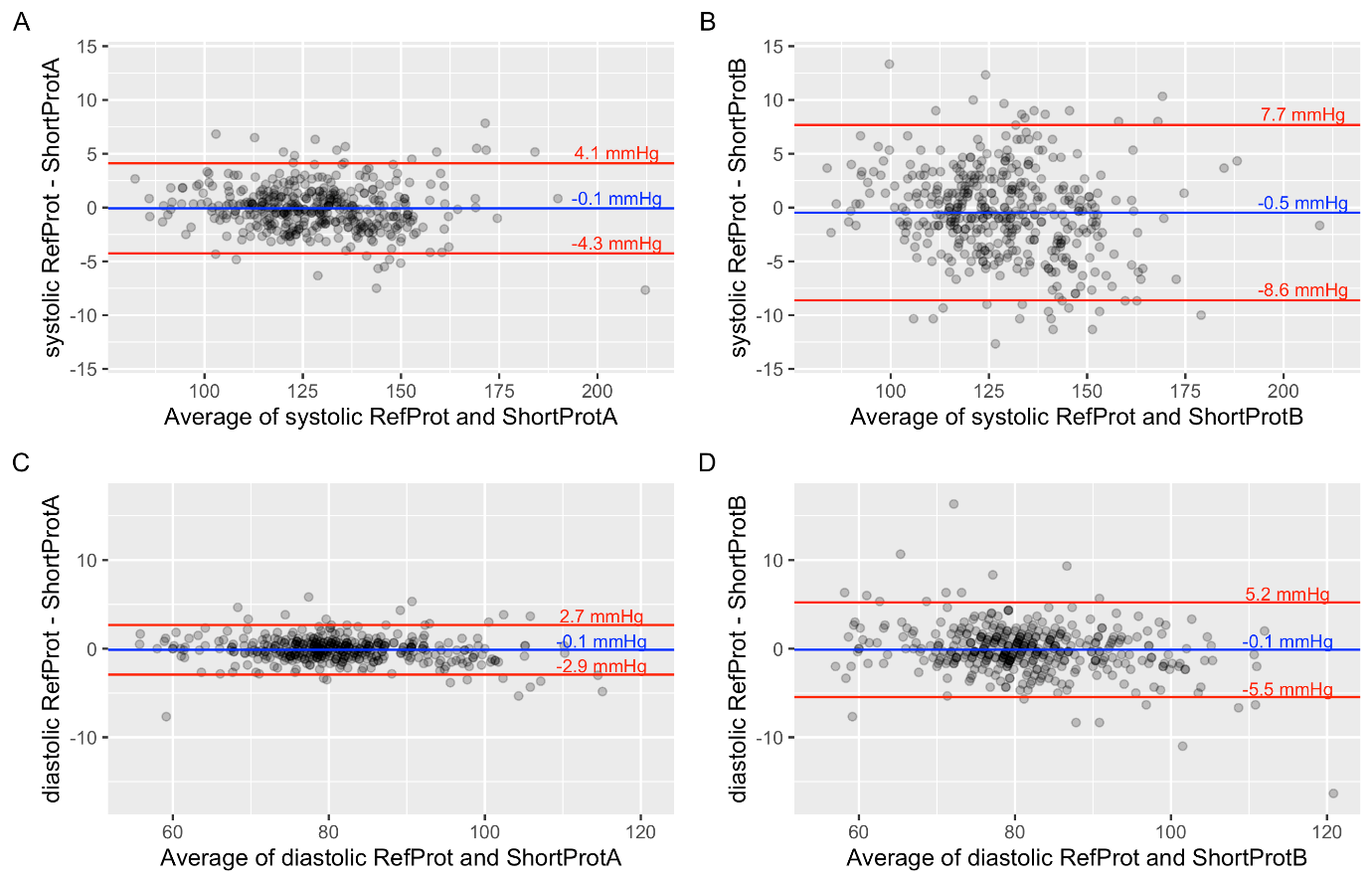
Every pair of comparison between RefProt and ShortProtA or ShortProtB is represented by an individual dot. Blue lines indicate mean difference; red lines the limits of agreement.
