# Supplementary material for: Accuracy of abbreviated protocols for unattended automated office blood pressure measurements, a retrospective study
Source: PLoS One. 2021 Mar 15;16(3):e0248586. doi: 10.1371/journal.pone.0248586 (PMC7959338; doi:10.1371/journal.pone.0248586)
Supplement: S3 Fig — One stethoscope equals to one AOBPM set. Pink: RefProt and ShortProt normotensive, Blue: RefProt normotensive, ShortProt hypertensive, Orange: RefProt hypertensive, ShortProt normotensive, Green: RefProt and ShortProt hypertensive. (DOCX) [file pone.0248586.s003.docx]

**Supporting Figure S3. Comparison of systolic BP classification of RefProt and ShortProtA (panel A), ShortProtB (panel B), and diastolic BP classification of RefProt and ShortProtA (panel C), and ShortProtB (panel D).**


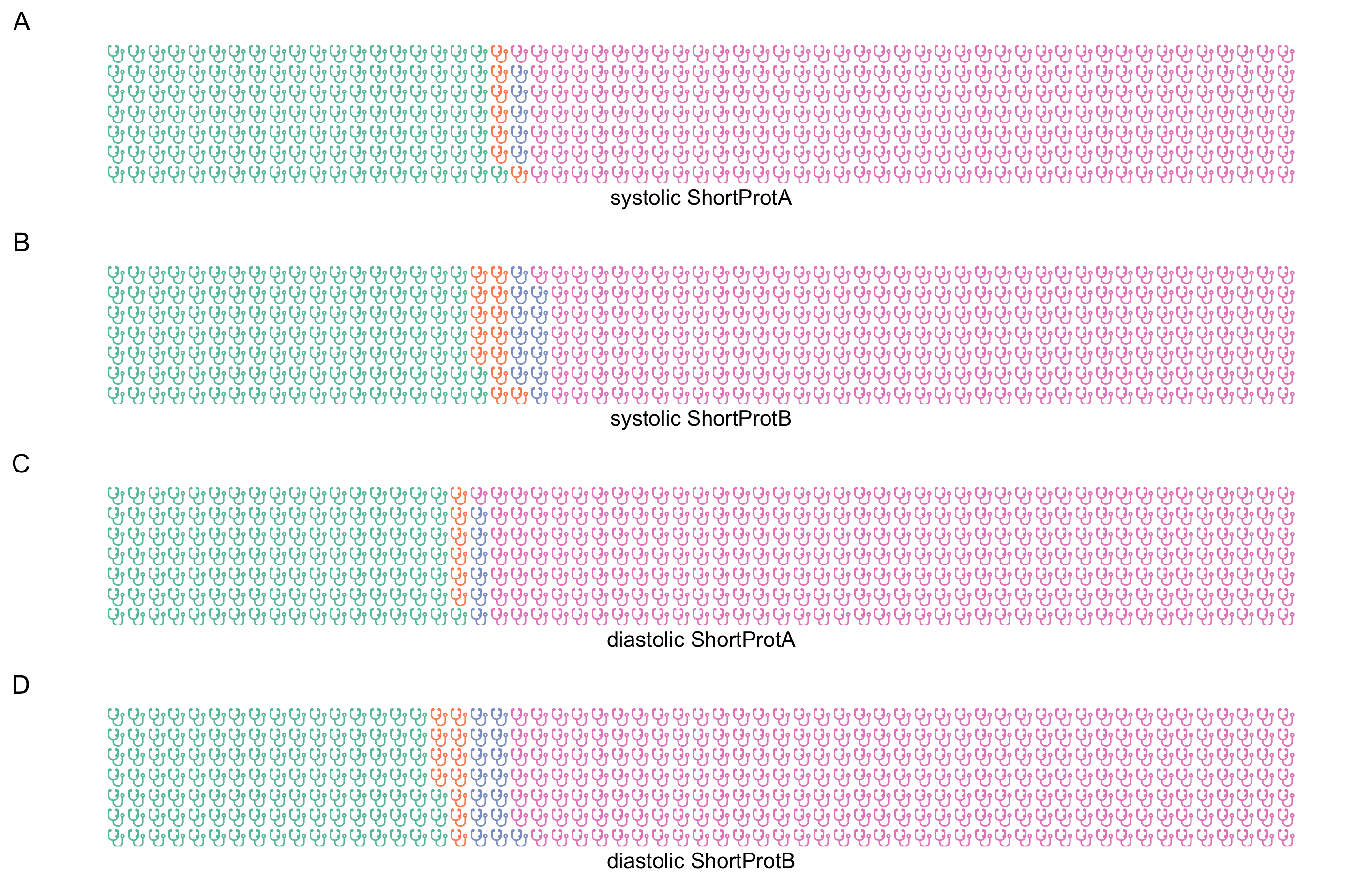
One stethoscope equals to one AOBPM set. Pink: RefProt and ShortProt normotensive, Blue: RefProt normotensive, ShortProt hypertensive, Orange: RefProt hypertensive, ShortProt normotensive, Green: RefProt and ShortProt hypertensive.
