# Supplementary material for: Accuracy of abbreviated protocols for unattended automated office blood pressure measurements, a retrospective study
Source: PLoS One. 2021 Mar 15;16(3):e0248586. doi: 10.1371/journal.pone.0248586 (PMC7959338; doi:10.1371/journal.pone.0248586)
Supplement: S1 Table — (DOCX) [file pone.0248586.s004.docx]

**Supporting Table S1: Baseline characteristics**

| **Parameter** | **N (total 210)** | **Median (IQR)** | **Mean (± SD)** |
| --- | --- | --- | --- |
| **Age, years** | NA | 51 (38 – 66) | 52 (± 16) |
| **Male sex** | 116 (55.2) | NA | NA |
| **Height, cm** | NA | 172.0 (164.0 – 178.0) | 171.5 (± 9.4) |
| **Weight, kg** | NA | 80.0 (69.5 – 93.5) | 82.5 (± 17.8) |
| **BMI, kg/m2** | NA | 27.4 (24.3 – 30.8) | 28.0 (± 5.6) |
| **Active smokers** | 36 (17.1) | NA | NA |
| **Previous smokers** | 55 (26.2) | NA | NA |
| **Pregnancy** | 10/94 (10.6) | NA | NA |
| **Comorbidities:** |  |  |  |
| **Arterial hypertension** | 201 (95.7) | NA | NA |
| **Diabetes mellitus** | 34 (16.2) | NA | NA |
| **Coronary artery disease** | 28 (13.3) | NA | NA |
| **Congestive heart failure** | 5 (2.4) | NA | NA |
| **Chronic kidney disease** | 34 (16.2) | NA | NA |
| **Peripheral artery disease** | 7 (3.3) | NA | NA |
| **TIA/Stroke** | 18 (8.6) | NA | NA |

Baseline characteristics shown as median (inter-quartile range (IQR)), mean (± standard deviation (SD)), or number (percentage), as appropriate. Comorbidities as reported by the patients. TIA = transient ischemic attack.
