# Supplementary material for: Accuracy of abbreviated protocols for unattended automated office blood pressure measurements, a retrospective study
Source: PLoS One. 2021 Mar 15;16(3):e0248586. doi: 10.1371/journal.pone.0248586 (PMC7959338; doi:10.1371/journal.pone.0248586)
Supplement: S2 Table — (DOCX) [file pone.0248586.s005.docx]

**Supporting Table S2: Number of measurements with an absolute difference <2, <5, <10 and <15 mmHg between the RefProt and ShortProtA and ShortProtB, respectively).**

| Absolute difference to RefProt | syst. ShortProtA  N (%) | syst. ShortProtB  N (%) | diast. ShortProtA  N (%) | diast. ShortProtB  N (%) |
| --- | --- | --- | --- | --- |
| < 2 mmHg | 344 (83.3) | 275 (66.6) | 381 (92.3) | 337 (81.6) |
| < 5 mmHg | 407 (98.5) | 353 (85.5) | 411 (99.5) | 400 (96.9) |
| < 10 mmHg | 413 (100) | 405 (98.1) | 413 (100) | 411 (99.5) |
| < 15 mmHg | 413 (100) | 413 (100) | 413 (100) | 412 (99.8) |

RefProt: mean of all three measurements. ShortProt: ShortProtA: mean of the first two measurements, ShortProtB: the single first measurement.
