# Supplementary material for: Accuracy of abbreviated protocols for unattended automated office blood pressure measurements, a retrospective study
Source: PLoS One. 2021 Mar 15;16(3):e0248586. doi: 10.1371/journal.pone.0248586 (PMC7959338; doi:10.1371/journal.pone.0248586)
Supplement: S3 Table — (DOCX) [file pone.0248586.s006.docx]

**Supporting Table S3: BP classification of ShortProt in comparison to RefProt.**

|  | **systolic** | | **diastolic** | |
| --- | --- | --- | --- | --- |
| **BP classification** | **ShortProtA**  **N (%)** | **ShortProtB**  **N (%)** | **ShortProtA**  **N (%)** | **ShortProtB**  **N (%)** |
| RefProt hypertensive, ShortProt hypertensive | 134 (32.4) | 128 (31.0) | 120 (29.1) | 115 (27.8) |
| RefProt hypertensive,  ShortProt normotensive | 7 (1.7) | 13 (3.1) | 6 (1.5) | 11 (2.7) |
| RefProt normotensive, ShortProt hypertensive | 5 (1.2) | 12 (2.9) | 6 (1.5) | 15 (3.6) |
| RefProt normotensive, ShortProt normotensive | 267 (64.6) | 260 (63.0) | 281 (68.0) | 272 (65.9) |
| P-value | 0.774 | 1.000 | 1.000 | 0.556 |
| Test statistic | 0.083 | 0.000 | 0.000 | 0.346 |

RefProt: mean of all three measurements. ShortProt: ShortProtA: mean of the first two measurements, ShortProtB: the single first measurement.
